# Selective Magnetic Nanoheating: Combining Iron Oxide Nanoparticles for Multi-Hot-Spot Induction and Sequential Regulation

**DOI:** 10.1021/acs.nanolett.1c02178

**Published:** 2021-08-19

**Authors:** Jesus G. Ovejero, Ilaria Armenia, David Serantes, Sabino Veintemillas-Verdaguer, Nicoll Zeballos, Fernando López-Gallego, Cordula Grüttner, Jesús M. de la Fuente, María del Puerto Morales, Valeria Grazu

**Affiliations:** † Institute of Materials Science of Madrid (ICMM-CSIC), Sor Juana Inés de la Cruz 3, 28049 Madrid, Spain; ‡ BioNanoSurf Group, Aragon Nanoscience and Materials Institute (INMA-CSIC-UNIZAR), Edificio I+D, Mariano Esquillor Gómez, 50018 Zaragoza, Spain; § Centro de Investigación Biomédica en Red de Bioingeniería, Biomateriales y Nanomedicina (CIBER-BBN), Avenida Monforte de Lemos, 3-5, 28029 Madrid, Spain; ∥ Applied Physics Department and Instituto de Investigacións Tecnolóxicas, Universidade de Santiago de Compostela, 15782 Santiago de Compostela, Spain; ⊥ Heterogeneous Biocatalysis Laboratory, Center for Cooperative Research in Biomaterials (CIC biomaGUNE), Basque Research and Technology Alliance, Paseo de Miramón 194, 20014 Donostia-San Sebastián, Spain; # IKERBASQUE, Basque Foundation for Science, María Díaz de Haro 3, 48013 Bilbao, Spain; ∇ Micromod, Partikeltechnologie GmbH, Friedrich-Barnewitz-Straße 4, 18119 Rostock, Germany

**Keywords:** Hot spot, Magnetic nanoparticles, Iron oxide, Thermal regulation, Local temperature, Nanothermometry, Molecular thermometers, Enzymes

## Abstract

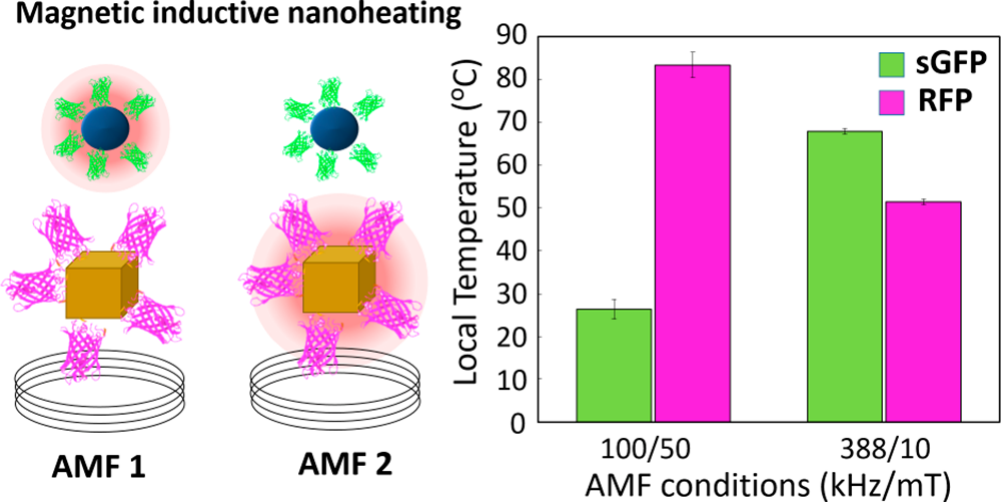

The contactless heating capacity of magnetic nanoparticles (MNPs) has been exploited in fields such as hyperthermia cancer therapy, catalysis, and enzymatic thermal regulation. Herein, we propose an advanced technology to generate multiple local temperatures in a single-pot reactor by exploiting the unique nanoheating features of iron oxide MNPs exposed to alternating magnetic fields (AMFs). The heating power of the MNPs depends on their magnetic features but also on the intensity and frequency conditions of the AMF. Using a mixture of diluted colloids of MNPs we were able to generate a multi-hot-spot reactor in which each population of MNPs can be selectively activated by adjusting the AMF conditions. The maximum temperature reached at the surface of each MNP was registered using independent fluorescent thermometers that mimic the molecular link between enzymes and MNPs. This technology paves the path for the implementation of a selective regulation of multienzymatic reactions.

## Introduction

The growing development of magnetic nanoparticles (MNPs) synthesis methods, especially iron oxide nanoparticles, has boosted their applicability on different fields such as biomedicine,^1^ water remediation,^2,3^ and nanocatalysis,^4^ among many other. These materials present unique advantages in terms of contactless manipulation, reusability, and biocompatibility, since iron oxide can be easily digested and integrated by bioorganisms.^5^ One of their most interesting features is the possibility of inducing local heat by irradiating them with alternating magnetic fields (AMFs). Besides, the MNPs can be prepared as magnetic colloids thanks to their lack of remanence (superparamagnetic regime) and being easily coated with biological components such as proteins or enzymes. These two features make MNPs ideal biocompatible nanoheaters.

The inductive heating power of the MNP colloids has been extensively applied to the thermal treatments of tumor cells^6^ and more recently to the regulation of enzymatic and catalytic processes.^7,8^ In contrast to other catalytic applications, the thermal regulation of an enzymatic activity or protein conformation mediated by MNPs requires an extreme control of the local temperatures achieved on the surface of the nanoheaters. The amount of heat dissipated depends on the MNP composition, size, shape, and aggregation state, but it depends also on the specific conditions (frequency and field) of the AMF applied.^9^ Tailoring these parameters, it is possible to optimize the local temperature (*T*_LOC_) induced in the surface of the MNPs to match different optimal operational temperatures (*T*_OPT_) of proteins or enzymes attached to their surface. Theoretical and experimental assays have shown that, although the temperature generated at the surface of the MNP can reach up to the boiling point of the media, it decays rapidly at a few nanometers from their surface.^10−12^ Through the preparation of diluted colloids of MNPs,^13^ Armenia et al. demonstrated that, taking advantage of this phenomenon, it is possible to create hot spots in the local environment of the enzymes enhancing their efficiency while maintaining the reactor temperature cold. This seminal work opened the gate to the creation of single-pot multienzymatic reactions operating simultaneously at different optimal temperatures or, alternatively, to the sequential activation of multienzymatic cascades by exploiting the versatility of MNPs as nanoheaters.

Currently, such a contactless magnetic heating regulation of enzyme activity has been restricted to the use of a single monodisperse population of MNPs with a homogeneous heating capacity. The idea of combining two MNPs populations with well-differentiated anisotropies to develop a selective system of thermal activation was first described by the theoretical studies of Anikeeva’s group in 2014.^14^ They recently applied this principle to the remote activation of heat-sensitive cation channels of kidney cells with outstanding results,^15^ and the tremendous potential of this technology can be exploited in many other fields such as tumor therapies.^16^ However, none of these studies analyze the specific *T*_LOC_ induced in the surface of each set of MNPs, which is a critical parameter in the case of biological transformations controlled by the biological activity of proteins including enzymes and an apoptotic induction of tumor cells.^17^

To analyze this effect, the use of temperature transducers directly linked to the surface of the MNP provides information about the temperature reached at the active position of the regulated protein during an AMF activation. The use of fluorescent molecules whose emission intensity depends on the temperature is a frequent strategy for local thermometry^18,19^ with several technological advantages with respect to other nanothermometry alternatives.^18,20^ Fluorescent proteins, such as the superfolder Green Fluorescent Protein (sGFP) or m-Cherry Red Fluorescent Protein (RFP), can be genetically engineered to be tagged with a 6xHis polypetide at their N-terminus in order to resemble a typical site-directed orientation link between enzymes and MNPs functionalized with divalent transition-metal coatings. These proteins suffer an irreversible unfolding denaturation with temperature that leads to a linear loss of fluorescence.^21,22^ Such linearity makes them interesting thermal probes for nanothermometry in intracellular^23−25^ and in vivo^26^ studies.

In this Communication, we demonstrate for the first time that, by using a well-designed toolbox of MNPs with different sizes and shapes, it is possible to generate a multi-hot-spot reactor in which the *T*_LOC_ may be adjusted by tuning the AMF conditions. For this aim, we developed a set of iron oxide nanoparticles with core sizes between 8 and 32 nm and different organic and polymeric coatings to create a set of magnetic nanoheaters with different heating powers and different optimum AMF conditions for heat dissipation. The global heating efficiency of the different cores and coatings was evaluated under AMFs between 5 and 60 mT and frequencies between 96 and 760 kHz. The surface of all these MNPs was engineered with different divalent copper-nitrile acetic acid (Cu^2+^-NTA) moieties, to selectively bind recombinant His-tagged variants of sGFP and RFP through a metal chelate affinity. These fluorescent proteins (FPs) were used as a biomolecular model to determine the maximum local temperature induced at the surface of MNPs when exposed to an AMF. In this way, we were able to measure and correlate the increment of global and local temperatures induced by the magnetic heating of MNPs and establish a versatile toolbox of magnetic nanoheaters that could match the requirements for a simultaneous or sequential activation of multicompenent biology systems in one pot.

## Results and Discussion

**Figure 1:**
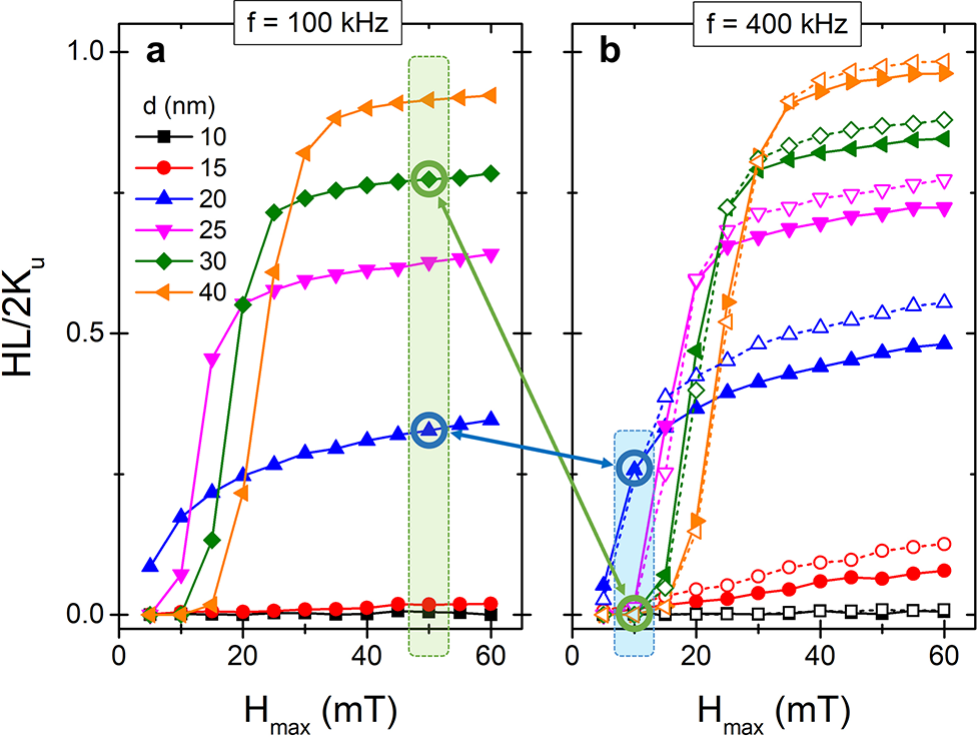
LLG macrospin simulations prove that the combination of high frequency–low field (blue square) and low frequency–high field (green square) AMFs offer an interesting mechanism to select the nanoheating activation. HLs normalized to the reduced anisotropy (*K*_u_) for MNPs of different sizes exposed to AMF of increasing *H*_MAX_ and fixed frequencies of (a) *f* = 100 and (b) 400 kHz. (b) The curves at 800 kHz were included as dash lines with open symbols. The highlighted sizes and field conditions illustrate how the alternate heat activation could be achieved.

The simplest strategy to modify the heating performance of the MNPs exposed to AMF is to modify their size. For a certain material, the anisotropy energy of the MNPs grows with the volume of the MNP as *E*_A_ = *K*_eff_*V*, with *K*_eff_ and *V* being the effective anisotropy energy constant and volume of the MNP, respectively. Figure 1 shows the theoretical dependence between the hysteresis losses (HL) in the MNPs and the maximum applied magnetic field (*H*_MAX_) at 100 and 400 kHz for a system of randomly distributed non-interacting monodisperse magnetite MNPs of increasing sizes at *T* = 300 K, with their magnetization *M⃗* being governed by the stochastic form of the Landau-Lifshitz-Gilbert (LLG) equation (see the Supporting Information for details).^27^

It can be observed that, independently of the size, the energy dissipated grows with the *H*_MAX_ following a sigmoidal dependence. The center and height of the sigmoid scale up with the size of the MNP. These graphs show how the large MNPs requires a higher *H*_MAX_ to produce a significant heat dissipation, but they achieve a higher dissipation power if the applied field is large enough. It can also be noticed that the saturation value for HL increases with the frequency of AMF, but the inflection points of the sigmoid curves suffer a minimum shifting at high frequencies (800 kHz in dash lines). Comparing the HL of intermediate (20 nm) and large MNPs (30 nm) at low and high frequencies, it is possible to extract a general strategy to choose AMF conditions that invert their heating power (blue and green boxes in Figure 1). Furthermore, the reduced anisotropy value (*K*_u_) presents a certain dependence with the nanoparticle size, which may add further possibilities for fine-tuning the heat release that has not been considered in the present simulations, particularly if working with particle sizes around and below the 10 nm range.^28,29^

On the one hand, below a certain threshold field (*H*_MAX_ = 15 mT for this selection) the MNPs of 30 nm do not transform the magnetic energy into heat losses, whereas the 20 nm MNPs can reach a theoretical limit of 570 W/g by increasing the AMF frequency to 400 kHz (Supporting Information, Figure S1). On the other hand, using an intense (50 mT) and low-frequency AMF (100 kHz) the MNPs of 30 nm result in better nanoheaters than the 20 nm ones, reaching a saturation value for HL equivalent to 430 W/g, while that of the 20 nm MNPs is reduced to 180 W/g. This high field–low frequency versus low field–high frequency (high H-low f vs low H-high f) strategy was first proposed by Anikeeva’s group as an AMF tuning parameter to select which MNPs is activated.^14^ Please note that the HL data have been plotted normalized by 2*K*_u_ (theoretical maximum for a randomly distributed system)^30^ to better illustrate the size effects on the heating performance. The corresponding specific absorption rate (SAR) data are shown in Figure S1, emphasizing the SAR difference due to the proportionality with frequency.

**Figure 2:**
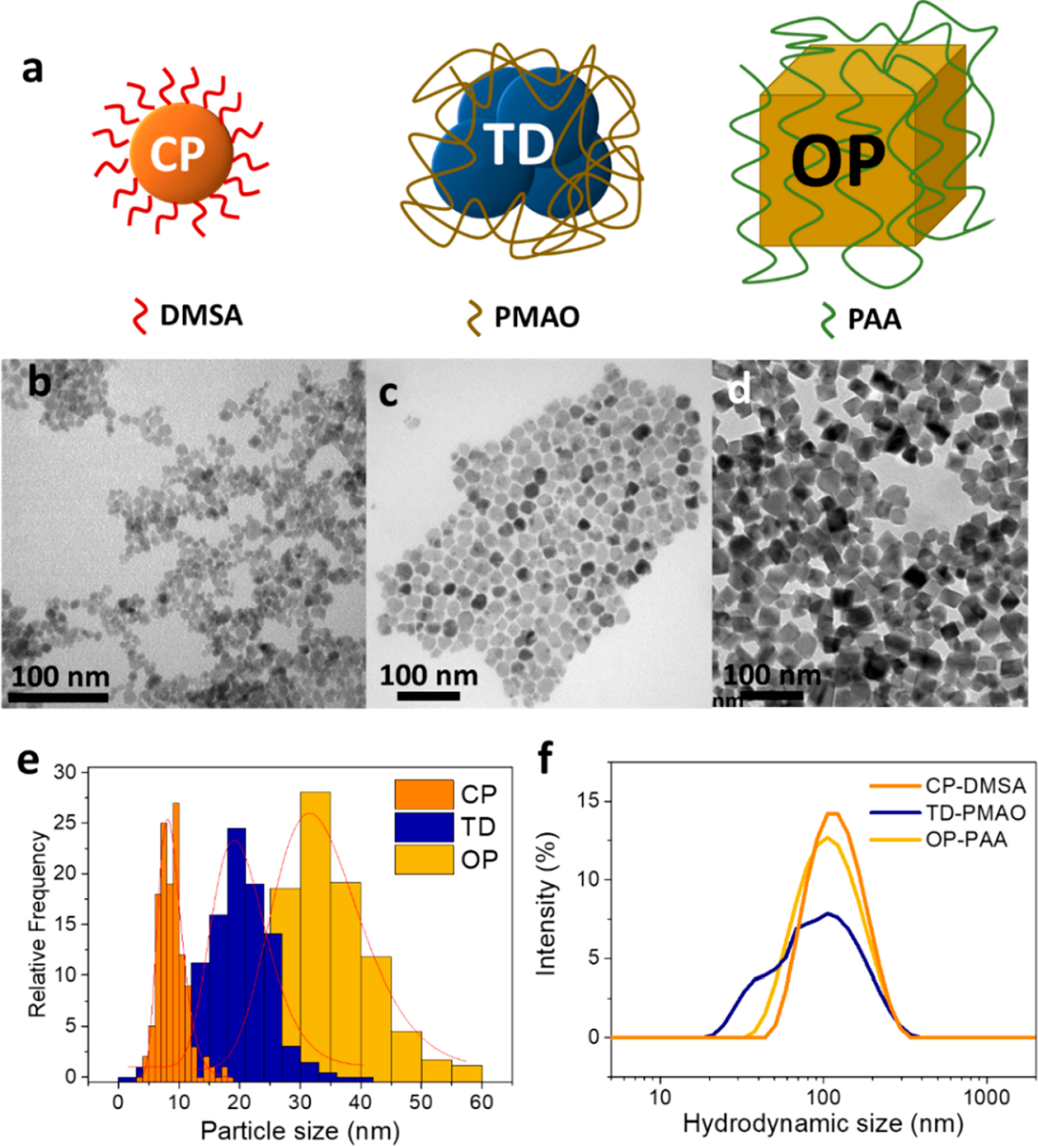
Three sets of MNPs were produced with different sizes and geometries. (a) Scheme of MNPs used as thermal regulators and the coatings used for stabilization. TEM pictures of MNPs prepared by (b) CP, (c) TD, and (d) OP. (e) TEM size distribution for the three sets of MNPs. Red curves indicate the log-normal fitting of the size distribution. (f) Dynamic light scattering intensity curves for the hydrodynamic size of the three sets of MNPs.

In the light of the theoretical results three monodisperse population of MNPs with average sizes ranging from 8.5 to 33.2 nm were prepared. To that aim, we performed three different synthesis methods to obtain a homogeneous distribution of MNPs with distribution widths (σ_TEM_) below 0.25 (Table S1). Figure 2 shows the transmission electron microscopy (TEM) images of the MNPs obtained by coprecipitation (CP), thermal decomposition (TD), and oxidative precipitation (OP). The CP synthesis generates spheroidal MNPs with an average size of 8.5 ± 2.0 nm. The MNPs prepared by TD present a larger average diameter (*D*_TEM_ = 20.2 ± 4.8 nm) and a multicore structure made of aggregates of smaller nanocrystals. The largest MNPs were obtained by OP. They present a tetrahedral geometry with an average size of 33.2 ± 7.9 nm. The X-ray diffraction patterns confirm an inverse spinel structure corresponding to magnetite/maghemite in all the cases and the polycrystalline structure of TD-MNPs (Figure S2). To generate a selective activation of a single population of MNP it is important that their average sizes are well-separated and that the widths of the size distributions are small. In this respect, it is of remarkable importance the small overlapping between the size distributions presented in Figure 2e.

The three systems were decorated with carboxylic groups in order to improve their colloidal stability and also introduce copper-nitrile acetic acid chelates (Cu^2+^-NTA) moieties onto the MNPs surface to ultimately coordinate the His-tagged fluorescent proteins. The CP MNPs were coated with a thin layer of dimercaptosuccinic acid (DMSA), whereas the larger MNPs prepared by TD and OP were stabilized with long charged polymers such as poly(maleic anhydride-*alt*-1-octadecene) (PMAO) and poly(acrylic acid) (PAA), respectively. These polymers introduce a steric barrier that ensures the colloidal stability of MNPs even when dispersed in saline buffers (Figure S3). Figure 2f shows the hydrodynamic size of the three systems after a surface coating. The samples present a principal hydrodynamic size of ∼100 nm that suggests the formation of primary aggregates made of a few MNPs during the coating (Table S1). In the case of the TD-PMAO sample, a secondary peak that appears at smaller hydrodynamic sizes indicates the presence of more individually coated MNPs.^31^ The proper coating of the MNPs was confirmed by thermogravimetric analysis, infrared spectroscopy, and Z-potential determination (Figure S4). Interestingly, the high-pressure coating protocol of OP-PAA produced a high-quality thin polymeric coating able to stabilize the MNPs with minimal polymeric content (3.4% of organic mass).

**Figure 3:**
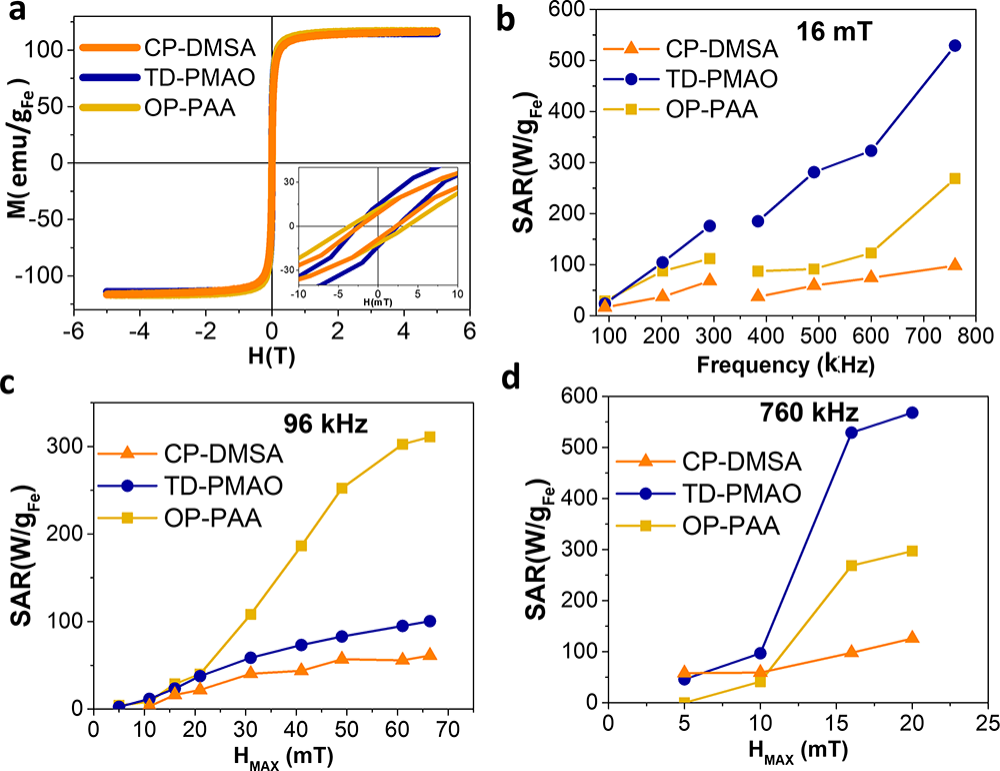
Specific magnetic features of each MNP generate a different heating power when exposed to AMFs. (a) Magnetization-field hysteresis loops of MNPs under a quasistatic condition. (inset) The central part of a cycle. (b) SAR of MNPs exposed to AMF of 16 mT at increasing frequencies. SAR vs *H*_MAX_ dependence of MNPs exposed to (c) low-frequency AMF (96 kHz) and (d) high-frequency AMF (760 kHz).

The magneto-thermal responses of the three systems were evaluated using quasistatic and AMFs. The hysteresis loops presented in Figure 3a show the magnetic cycle under quasistatic conditions. All of them present a maximum magnetization at ∼105 ± 2 emu/g_Fe_, which is consistent with maghemite saturation magnetization,^32^ but important differences can be appreciated in the low-intensity field range inset. The CP-DMSA and TD-PMAO samples present a similar coercivity (*H*_C_ = 2.5 mT). However, the collective magnetic behavior of the nanocrystals inside the TD-PMAO nanoparticle increases significantly the susceptibility of the cycles.^33,34^ In the opposite extreme, the hysteresis loop of the OP-PAA sample presents larger coercivity (*H*_C_ = 3.75 mT) and smaller susceptibility.

The different magnetic response between the three samples implies a different heating power when exposed to an AMF. The amount of heat dissipated is generally expressed by an empirical parameter called SAR that quantifies the amount of heat transmitted to the medium.^9^Figure 3b shows that, at low-intensity AMF (*H*_MAX_ = 16 mT), the TD-PMAO sample generates a larger SAR in the whole range of frequencies studied, whereas the SAR is always the minimum for CP-DMSA. By an analysis of the SAR versus *H*_MAX_ curves presented in Figure 3c,d, the sigmoidal dependence predicted by the theoretical models can be identified in the three samples at low and high frequencies. It can be observed that the TD-PMAO and OP-PAA are interesting systems to exploit the selective activation strategy based on a high H–low f versus low f–high H strategy.

**Figure 4:**
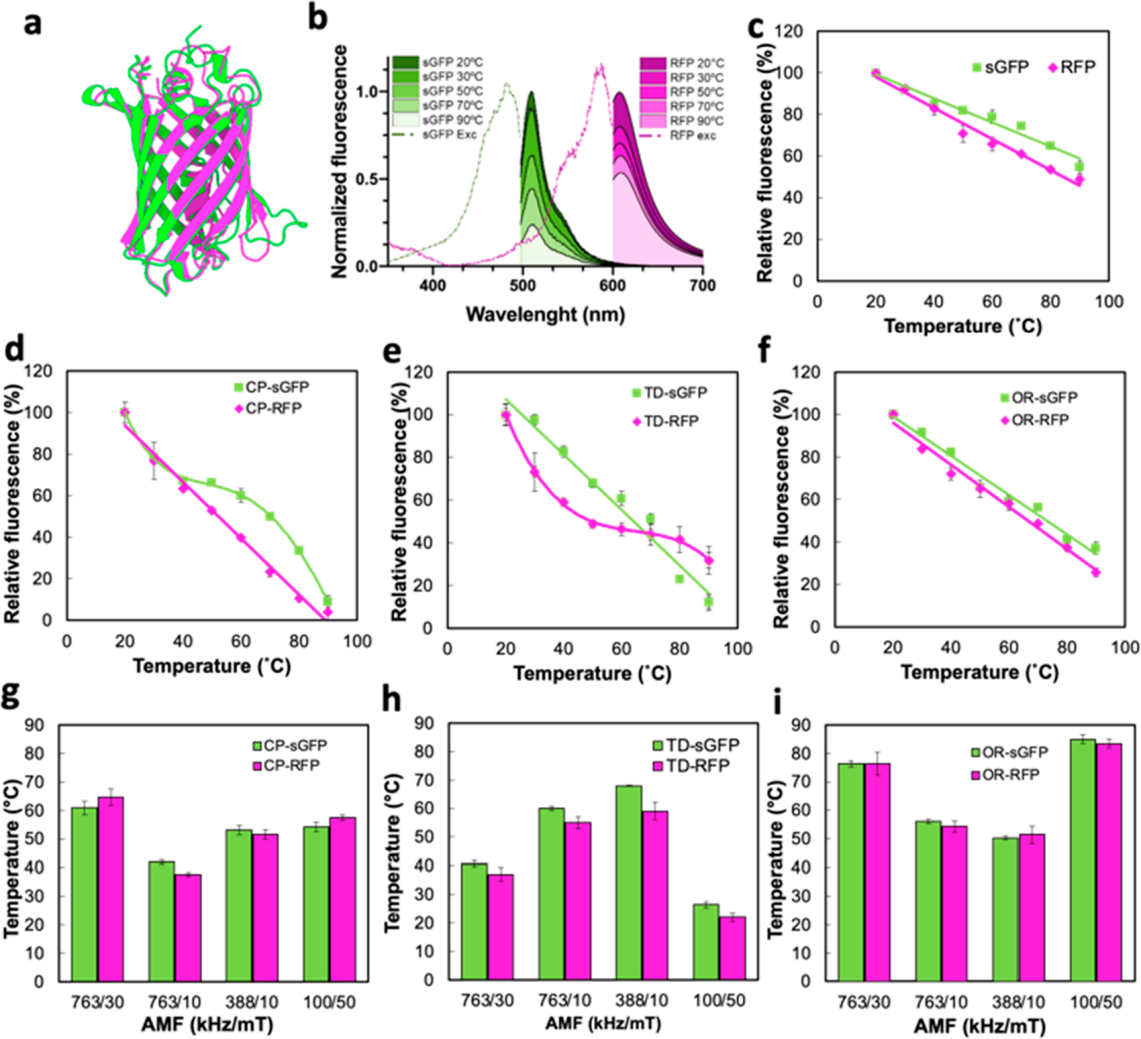
sGFP and m-Cherry RFP were used as a thermal probe of the local temperature in the environment of MNPs. (a) Superimposed representation of a three-dimensional structure of the ternary structure of sGFP and RFP (visualized using Protein Imager^42^). (b) Intensity reduction of sGFP (green) and RFP (magenta) fluorescence spectra with temperature applied using a global heating source (thermoblock). Dash lines indicate their respective absorption spectra at 20 °C. (c) Relative fluorescence intensity of free sGFP and RFP proteins at increasing temperatures. Relative fluorescence intensity of sGFP and RFP eluted from the surface of (d) CP, (e) TD, and (f) OP after 5 min of incubation in a thermoblock. Estimated local temperature (*T*_LOC_) registered from fluorescence loss sGFP and RFP eluted from (g) CP, (h) TD, and (i) OP complexes exposed to different AMF conditions for 5 min.

The *T*_LOC_ induced by the AMF heating was studied using the above-mentioned MNPs conjugated with two different recombinant his-tagged fluorescent proteins, namely, sGFP and RFP. These two proteins present a similar β-barrel tertiary structure displayed in Figure 4a, but their different fluorophore centers generate fluorescence spectra with well-separated emission peaks (Figure 4b).^35,36^ The tertiary structure of both proteins is affected by the temperature suffering the loss of their fluorescence intensity. Besides, Figure 4c shows that, in both cases, the fluorescence of soluble proteins decays linearly as the temperature increases between 20 and 90 °C. The higher reduction observed in sGFP may be attributed to the higher stability of the resonant chain in RFP.^37^

The conjugation of the proteins with inorganic substrates may lead to changes and/or a rigidification of their structure that modifies their temperature stability.^38^ To analyze the effect of temperature on the fluorescence intensity of the proteins grafted to the MNPs surface, the fluorescent proteins were eluted in the presence of 0.5 M imidazole and segregated from the MNPs by an ultracentrifugation after the thermal treatments. The fluorescence versus temperature curves presented in Figure 4d–f for MNP-sGFP and MNP-RFP conjugates reveal a loss of linearity for CP-sGFP and TD-RFP samples, while OP MNPs fluorescent complexes preserved a linear dependence for the two tested proteins. As expected, the interaction between fluorescent proteins and the MNPs substrates alters its thermal stability in different ways depending on the nature of the coating and the interactions formed at the MNPs-protein interphase during protein binding. Biphasic dependences of the fluorescence with a temperature like those observed for CP-sGFP and TD-RFP are usually observed when the fluorophores present two light-emitting states,^39^ as in the case of sGFP and RFP.^36,40^ The transition between these two states depends on the conformation of the nearest amino acids to the chromophore and may be affected by the interaction with MNP coating.^41^

In all the complexes, the immobilization of FPs on the three MNPs drives to less thermally stable protein as observed from the higher slope of the fluorescence versus *T* curves. Such a reduction in the thermal stability of the proteins may represent an advantage in the case of local nanothermometry. The higher slope of the intensity versus temperature curves translates into a higher sensitivity to the *T*_LOC_ when used as a thermometer. The linear and polynomial fittings presented as continuous lines in Figure 4d–f were used as calibration curves to estimate the maximum *T*_LOC_ achieved at the protein position during magnetic heating experiments. Table S3 collects the slopes of the linear fittings and the polynomial parameters used for the fitting of nonlinear curves.

Figure 4g–i shows the *T*_LOC_ registered from the fluorescence of sGFP and RFP after exposing MNPs-sGFP and MNPs-RFP complexes to AMFs with different conditions of frequency and field for 5 min. The temperature registered in the media remained constant at 17 ± 1 °C through all AMF exposure indicating that the inductive heating was constrained to the local environment of the MNPs due to the low concentrations of the colloids (5 μg_Fe_/mL). The independent measurements of *T*_LOC_ obtained from sGFP and RFP nanothermometers conjugated to the three types of MNPs present a significant congruence between them despite the differences observed in their calibration curves. The results probe the robustness and versatility of the thermometric system proposed.

Besides, the results obtained from local thermometry are in good agreement with the SAR values presented in Figure 3, once the specific features of each complex are taken into account. The smallest complexes (CP) generate a *T*_LOC_ between 50 and 60 °C independently on the AMF conditions applied. The SAR values of these particles are also the smallest (<75 W/g) for the AMF conditions explored, but thanks to the close proximity of the molecular thermometers to the surface of the MNPs they reach a moderate *T*_LOC_ in every AMF condition. In the case of TD complexes, a little increment of *T*_LOC_ was registered when exposed to a low-frequency AMF (AMF_1_ = 100 kHz to 50 mT). The polymeric coating of this sample introduces a thick spacer between MNPs surface and the molecular thermometer. Only when the AMF conditions are highly favorable (AMF_2_ = 388 kHz to 15 mT) does the system reache a *T*_LOC_ of ∼70 ± 5 °C at the protein position. In the case of OP complexes, the *T*_LOC_ observed at AMF_1_ is between 80 and 90 °C and decays to 50–60 °C for AMF_2_. In this sample, the thin PAA coating implies a closer proximity of the thermometer to the surface of the MNPs. This result highlights the importance of controlling the NTA-His-tag bindings and coatings thicknesses to predict the *T*_LOC_ induced in the protein position.^13^

**Figure 5:**
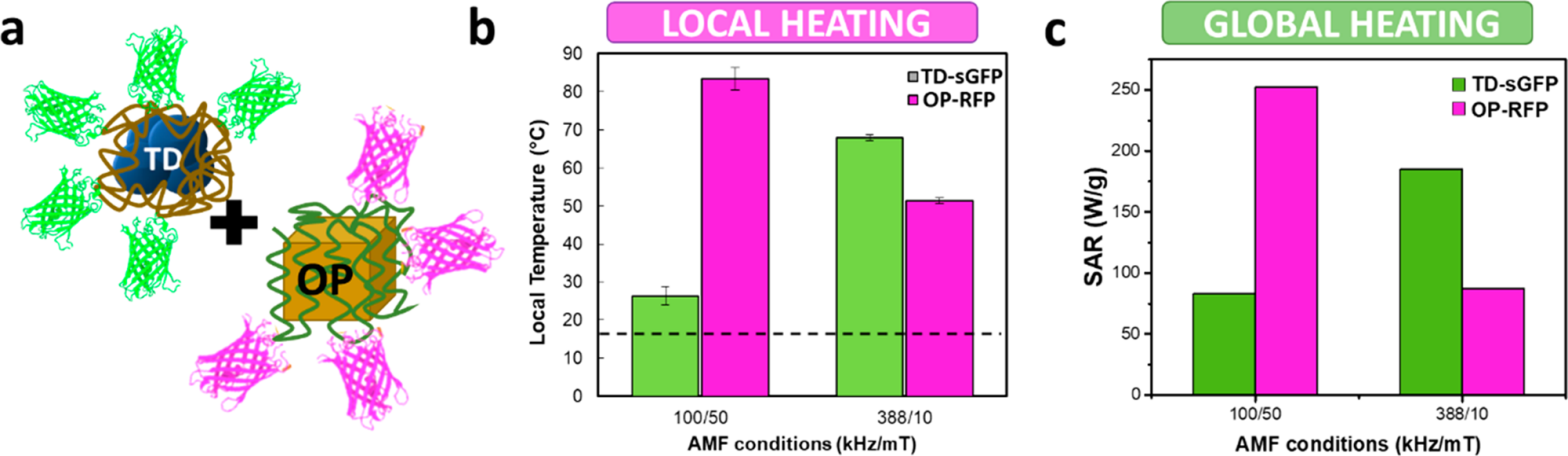
(a) With a mixture colloid of OP-RFP and TD-sGFP complexes it is possible to induce multiple hot spots in the same reactor and adjust each *T*_LOC_ by tuning the AMF conditions. (b) Estimated local temperature (*T*_LOC_) registered from sGFP and RFP fluorescence in the mixture colloid after 5 min of exposure to AMF_1_ = 100 kHz to 50 mT and AMF_2_ = 388 kHz to 10 mT. Black dash line indicates the global temperature registered in the medium. (c) SAR registered for individual concentrated colloids (1 mg/mL) of OP-RFP and TD-sGFP at AMF_1_ and AMF_2_.

The potential of MNPs to create a selective heating reactor was evaluated by mixing in a single pot TD-sGFP and OP-RFP complexes. For this experiment a CP sample was excluded due to its weak dependence of T_LOC_ with the AMF conditions, in the range explored. Figure 5 presents *T*_LOC_ registered by fluorescence nanothermometry when the mixed suspension is exposed to AMF_1_ (100 kHz/50 mT) and AMF_2_ (388 kHz/10 mT) conditions. The temperatures registered by each nanothermometer match with those observed in individual colloids (Figure 4), confirming the locality of the heat dissipation processes and thermal independence of each system of MNPs.

Furthermore, the temperatures registered at the two AMF conditions prove that this combination of complexes is suitable to perform a simultaneous multihot-spot and a sequential activation of enzymes. Using AMF_1_, it is possible to create a *T*_LOC_ of 25 ± 5 °C at the surface of TD-sGFP and 85 ± 5 °C at the surface of OP-RFP in a reactor that maintains its global temperature at 17 ± 1 °C. In contrast, by with AMF_2_, the *T*_LOC_ of TD-sGFP rose to 70 °C, and the *T*_LOC_ of OP-RFP was reduced to 50 °C. Figure 5c shows that the *T*_LOC_ registered in diluted colloids of each kind of MNP correlate with their heating power registered in higher concentrations (1 mg/mL).

The use of two independent fluorescent thermal probes for the analysis of multi-hot-spots formed in a pot is a landmark for local nanothermometry. Dual color fluorescence has been previously used for the analysis of mixtures of biological species to characterize their interactions^43^ and is a common protocol in ratiometric fluorescence thermometry.^44,45^ But, to the best of our knowledge, we pioneer their use to determine the local temperatures induced in a mixture of local nanoheaters activated by a common AMF. In contrast to the common fluorescence microscopy, this approach measures the local temperatures obtaining the fluorescence signal for the whole mixture colloid avoiding any selective imaging bias.^44,46^

## Conclusions

Using a clever combination of iron oxide magnetic nanoparticles and local thermal probes based on fluorescent proteins we have proved that it is possible to create both sequential and simultaneous multi-hot-spot conditions with different *T*_LOC_ in a single pot using different AMF settings. The selection of an adequate combination of magnetic nanoparticles requires a careful control of the magnetothermal properties and homogeneity of magnetic nanoheaters but also a precise control on the arrangement of active proteins on their surface. With diluted colloids, it is possible to heat selectively the environment of the nanoparticles maintaining a low global temperature in the dispersing media. The specific features of the magnetic nanoparticles can be tailored to obtain an optimum heating performance at a specific alternating magnetic field. This technology may create a new paradigm in the regulation of biological molecules such as the creation of one-pot multienzymatic cascades operating at multiple optimal temperatures or being sequentialy activated with different magnetic fields.

## Supporting Information Available

Experimental section with theoretical and empirical method details. Complementary data about theoretical calculations of SAR; X-ray diffraction patterns of uncoated MNPs; colloidal parameters and stability of the three MNPs; coating characterization (TGA, FT-IR, and Z-potentials); fluorescent proteins physicochemical paramenters and stability analysis; fitting parameters for thermocalibration curves; hydrodynamic sizes of MNP-FP complexes (PDF)

## Abbreviations

MNPs: Magnetic Nanoparticles
AMF: Alternating Magnetic Fields
TLOC: Local temperature
HL: Hysteresis losses
HMAX: Maximum Applied Field
HC: Coercive field
SAR: Specific Absorption Rate
CP: Coprecipitation
TD: Thermal decomposition
OP: Oxidative precipitation
NTA: Nitrile Acetic Acid
DMSA: Dimercaptosuccinic acid
PMAO: Poly(maleic anhydride-*alt*-1-octadecene)
PAA: Poly(acrylic acid)
sGFP: superfolder Green Fluorescent Protein
RFP: m-Cherry Red Fluorescent Protein
