# Supplementary material for: Selective Magnetic Nanoheating: Combining Iron Oxide Nanoparticles for Multi-Hot-Spot Induction and Sequential Regulation
Source: Nano Lett. 2021 Aug 19;21(17):7213–20. doi: 10.1021/acs.nanolett.1c02178 (PMC8431726; doi:10.1021/acs.nanolett.1c02178)
Supplement: Supplementary file 1 — nl1c02178_si_001.pdf [file nl1c02178_si_001.pdf]

Supporting Information: Selective Magnetic Nanoheating: Combining Iron Oxide Nanoparticles for Multi-hot-spot Induction and Sequential Regulation

Jesus G. Ovejero[1]*, Ilaria Armenia[2], David Serantes[4], Sabino Veintemillas-Verdaguer[1], Nicoll Zeballos[5,6], Fernando López-Gallego[5,6], Cordula Grüttner[7], Jesús M. de la Fuente[2,3], María del Puerto Morales[1] and Valeria Grazu[2,3]*

[1] Institute of Materials Science of Madrid (ICMM-CSIC), Sor Juana Inés de la Cruz 3, 28049 Madrid, Spain.

[2] BioNanoSurf Group, Aragon Nanoscience and Materials Institute (INMA-CSIC-UNIZAR), Edificio I+D, Mariano Esquillor Gómez, 50018 Zaragoza, Spain.

[3]Centro de Investigación Biomédica en Red de Bioingeniería, Biomateriales y Nanomedicina (CIBER-BBN), Avenida Monforte de Lemos, 3-5, 28029 Madrid, Spain.

[4]Applied Physics Department and Instituto de Investigacións Tecnolóxicas, Universidade de Santiago de Compostela, 15782 Santiago de Compostela, Spain.

[5]Heterogeneous Biocatalysis Laboratory, Center for Cooperative Research in Biomaterials (CIC biomaGUNE), Basque Research and Technology Alliance (BRTA), Paseo de Miramón 194, 20014 Donostia-San Sebastián, Spain.

[6]IKERBASQUE, Basque Foundation for Science, María Díaz de Haro 3, 48013 Bilbao, Spain.

[7]Micromod, Partikeltechnologie GmbH, Friedrich-Barnewitz-Straße 4, 18119 Rostock.

Experimental Section

Computational details

The behavior of a monodisperse system of non-interacting system of magnetic nanoparticles was simulated under the macrospin approximation by using the OOMMF software package. The procedure followed was the same as in Ref. [1]: each particle was associated to a unit cubic cell of volume V (with neither intercell exchange nor demagnetizing fields), and characterized by its magnetic anisotropy and magnetic *supermoment*, $\mu = M_S V$, where V is the particle volume and M_S is the saturation magnetization, taken as 420 emu/cm^3. For each particle (unit cell) two different anisotropies where considered: the intrinsic magnetocrystalline one, taken as that of magnetite (K_C = -11 kJ/m^3), and an additional uniaxial term of value K_u = 10 kJ/m^3. Both anisotropy terms were taken as uncorrelated, with their easy axes randomly distributed in space.

The dynamics of the magnetization was described by the Landau-Lifshitz-Gilbert equation with a random field to account for the thermal effects as presented in Equation 1[2].

$$\frac{d\vec{M}}{dt} = \frac{-\gamma}{1+\alpha^2}\vec{M} \times \left(\vec{H}_{eff} + \vec{H}_{th}\right) - \frac{\alpha\gamma}{M_S(1+\alpha^2)}\vec{M} \times \left[\vec{M} \times \left(\vec{H}_{eff} + \vec{H}_{th}\right)\right] \qquad (1)$$

Where γ is the electron gyromagnetic ratio, α the damping coefficient, M_S the saturation magnetisation, and $\vec{H}_{eff}$ and $\vec{H}_{th}$ are, respectively, the effective magnetic field acting on the particles and a random field that accounts for thermal effects. In the present simulations we have considered $\alpha = 0.1$, $M_S = 420$ emu/cm^3, and K_{eff} to be comprised of both the dominating uniaxial term $K_u = 1.1 \cdot 10^5$ erg/cm^3 and the intrinsic magnetocrystalline cubic anisotropy constant $K_C = -1.1 \cdot 10^5$. The results were averaged over 1000 particles over several cycles, to ensure the absence of transient behavior.

Preparation of MNPs by coprecipitation in aqueous media

Small MNPs were synthesized by Massart coprecipitation protocol [3]. In short, 445 mL of a mixture of $FeCl_3{\bullet}6H_2O$ (0.09 mol) and $FeCl_2{\bullet}4H_2O$ (0.054 mol) were added to 75 ml of NH_4OH (25%). The ferrous salt solution was slowly poured in the base at room temperature with vigorous stirring. To enlarge the size of MNPs prepared by this method, the mixture was heated up to 90ºC for 1 h and left to cool down to room temperature. The final product (aprox. 10 g) was washed three times with distilled water by magnetic decantation.

To complete the oxidation of MNPs prepared by coprecipitation and oxidative precipitation and to obtain a single phase of maghemite (γ-Fe_2O_3), both samples were treated with HNO_3 and $Fe(NO_3)_3$. This procedure, referred as acid treatment, is based on an oxidation-dispersion process previously reported [4]. The MNPs were treated with 300 mL of HNO_3 (2 M) for 15 min. Then, nitric acid was removed by magnetic decantation, and 75 mL of $Fe(NO_3)_3$ (1 M) and 130 mL of water were added. The mixture was heated up to 90ºC and stirred for 30 min. The particles were then cooled to room temperature, and separated with a permanent magnet.

The supernatant was decanted and substituted by 300 mL of HNO_3 (2 M) for 15 min. Finally, the MNPs were washed three times with acetone and redispersed in water. A rotary evaporator was used to remove any acetone contamination and concentrate the sample.

MNPs prepared by coprecipitation were coated with dimercaptosuccinic acid (DMSA) using an aqueous approach[5]. 5 mg (0.027 mol) of DMSA were added dissolved in 5 mL of deionized water and mixed at 50ºC under vigorous stirring. The DMSA solution was added to a suspension of 5 mL of MNPs (8.6 g Fe_2O_3/L) at pH 3 and sonicated for 1 minute. The pH of the mixture was raised to 11 and sonicated for 20 min. After this time, the sample was dialyzed for 48 hours.

Preparation of MNPs by thermal decomposition

Magnetic nanoparticles were obtained by decomposition of Fe(III) acetylacetonate (100 mM) in dibenzyl ether (1 L) in the presence of oleic acid (300 mM) and 1,2 dodecanediol (200 mM) under nitrogen flow (9 L/min) and stirring at 100 rpm. The mixture was heated up to 200ºC at 3ºC/min and kept for 2 h. Then, the temperature was increased up to the boiling point (295ºC) at 6ºC/min and kept for another hour. The reaction was stopped by removing the mantle and leaving the mixture to cool down. The washing process consists of precipitating the nanoparticles with a mixture of hexane/ethanol (1:3) vol (final volume 2500 mL) and discarding the supernatant containing by-products, surfactants and rest of solvents after two days of magnetic separation. Three washing steps with 150 mL of toluene/ethanol (1:2) vol leaded to the magnetite/maghemite final product (6.2 g) which was redispersed in 170mL of a solution of oleic acid in toluene 13%wt using an ultrasound bath (15min).

To transfer these MNPs to water, their coating with (PMAO) was carried out according to the protocol reported by Moros et al. was carried out[6]. Briefly, 240 mg of poly(maleic anhydride-alt-1-octadecene) (PMAO, MW: 30000-50000 Da) were added to a 500 mL flask containing 198 mL of chloroform. Then 20 mg of the hydrophobic nanoparticles (previously washed three times with ethanol and resuspended in 2 mL of chloroform) were added drop-wise in an

ultrasonic bath and the mixture was kept in the bath for 15 minutes at room temperature. Then the most part of the chloroform was removed under vacuum leaving a volume of 5-10 mL solution. 10 mL of Milli-Q water and 10 mL of 0.1 M NaOH were added at once (foaming occurs due to the hydrolysis of the maleic anhydride groups) and the solvent was removed at 70º C increasing the vacuum progressively until the complete disappearance of foam. The solution was then filtered using a 0.2 µm syringe filter. Finally, four ultracentrifugation steps were carried out during 2 hours at 24000 rpm at 15°C to eliminate unbound polymer.

Preparation of MNPs by oxidative precipitation in aqueous media

Large MNPs with cubic shape were prepared by a direct method in aqueous medium called oxidative precipitation[7]. Briefly, a 1M solution of $FeSO_4$ was prepared dissolving 13.90 g $FeSO_4$•$7H_2O$ in 50 mL of H_2SO_4 0.01M. The ferrous solution was quickly added to a basic solution prepared with 4.25 g of $NaNO_3$ and 4.22 g of NaOH in a mixture of 137 mL of water and 63 mL of ethanol 96% vol. The suspension of green rust obtained was stirred for 15 min and poured in a jacketed flask previously thermalized to 90 ºC with an oilthermostatic bath. The MNPs were left to grow at 90ºC for 6 hours. The whole process was performed in a glove box under nitrogen atmosphere to avoid the formation of secondary Fe oxide phases. The final precipitate was washed with DI water by magnetic decantation obtaining 3.8g of magnetite/maghemite nanoparticles.

MNPs prepared by this route were subjected to an acid treatment already described for the coprecipitation particles and then, coated by Micromod with polyacrylic acid sodium salt (PAA-Na) (1.2 kDa) under high pressure homogenization conditions, following a procedure described before in a patented process[8].

NTA-Cu^{2+} complex preparation

For this step, a modified nitrile acetic acid (NTA) molecule which contains an extra tail ending in a primary amine group (NH_2) was used. NTA-Cu^{2+} preparation was performed by preparing a suspension containing 45 mM of Nα- Nα- Bis(carboxymethyl)-L-lysine hydrate (Sigma

Aldrich, USA) and 50 mM of copper sulphate. The pH of the solution was adjusted to 10.5 using sodium hydroxide (Sigma Aldrich, USA). The solution obtained was centrifuged at 4000·g for 10 minutes to eliminate the unreacted salt. The supernatant was collected, and its pH was adjusted to 8.5-8.9.

NTA-Cu^{2+} functionalization of MNPs

To functionalize the CP and OP MNPs, a solution containing 1-(3-Dimethylaminopropyl)-3-ethylcarbodiimide (EDC - Sigma Aldrich, USA) and N-Hydroxysulfosuccinimide (s-NHS - Sigma Aldrich, USA) was prepared, added to a volume of particles corresponding to 1 mg_{Fe} and the volume was adjusted to 1 mL. After incubation at room temperature for 10 min, 500 µL of NTA-Me^{2+} was added[9]. The obtained suspension was incubated at room temperature for 2 hours. Then a solution of α-Methoxy-ω-amino PEG of 5000 Da (RappPolymer, Germany) was poured into the suspension to block the unreacted carboxylic groups and to increase the colloidal stability of the particles. After 45 minutes of stirring at room temperature, the particles were separated with a magnet or by ultra-centrifugation at 4000 x g for 5 minutes. The obtained particles were suspended in fresh buffer and stored at 4°C. The supernatant of the reaction was collected and stored at 4°C for further analysis.

In the case of TD MNPs, NTA and $CuSO_4$ were incubated in 500 µL of SSB for 5 minutes and centrifuged at 4500 rpm for 5 minutes. The supernatant was collected and added to a solution containing PEG 750 Da. 1 mg of PMAO functionalized MNPs were added to the solution above. Then the EDC was suspended in 20 µL and added to the suspension and stir at room temperature and protected from light. Then the same amount of EDC was added to the suspension. The mixture was stirred for three hours at room temperature or at 37°C and protected from light. To eliminate the excess of reagents several washing steps with Milli-Q water by centrifugation (5000 rpm for 15 min, 2 times) were carried out using 4 mL cellulose membrane centrifugal filters (Amicon, MilliPore, 100 kDa). The particles were recovered from the filters using Milli-Q water and stored at 4 ºC.

Structural characterization

The size and morphology of the particles were determined by transmission electron microscopy (TEM, JEOL JEM 1010) operating at 100.0 keV. The size distributions of samples were determined measuring more than 150 MNPs with ImageJ digital software and adjusting the results with a log-normal fitting of two free variables: mean (D_{TEM}) and distribution width (σ_{TEM}). Crystalline structures were determined by X-ray diffraction using Bruker D8 Advance diffractometer with a Cu Kα radiation lamp in Bragg-Brentano NTAconfiguration. The crystal size was obtained from the width of (311) spinel peak using Scherrer equation. Hydrodynamic size and Z-potential were determined with a Malvern Instrument Zetasizer Nano ZS (Malvern, UK) operating at 633 nm. Fe concentration in the colloids was determined by inductively coupled plasma optical emission spectroscopy (ICP-OES, ICP PERKIN ELMER mod. OPTIMA 2100 DV), after digesting a controlled volume of the sample (25 µL diluted to 25 mL). Fourier-Transformed spectra were measured in a Bruker IFS 66VS with a spectral range of 400 – 4000 cm^{-1}. The samples were dried and mixed with KBr to create a compacted pellet. Thermogravimetric curves (ATD/DSC/TG, Q600 TA Instruments) were obtained from dried samples heating from room temperature to 900°C in N_2 atmosphere at a heating rate of 10°C/min.

Magnetic and calorimetric characterization

Magnetic characterization was carried out in a Vibrating Sample Magnetomerter (MagLabVSM, Oxford Instrument). 50 µL of MNPs were dropped in a cotton pellet and dried in an oven at 50°C overnight. The dried cotton was compacted in a standard gelatine capsule. The magnetization values were normalized to the amount of Fe in the volume dried determined by ICP-OES.

Low frequency calorimetric measurements (96-286 kHz) were carried out in an AMF inductor (Fivesceles, MP 6kW) with an optic fibre thermal probe (OSENSA Innovations) introduced in

the sample holder. High frequency calorimetric measurements (388-763 kHz) were carried out in an AMF inductor D5 Drivers (nB nanomagnetics). Non-adiabatic heating curves were acquired by irradiating 1 mL of MNPs colloid with and Fe concentration of 1 g L^{-1} with two AMF conditions: 100 kHz-50 mT and 300 kHz-15 mT.

Heating power of MNPs was quantified using the specific absorption rate (SAR) obtained from equation 2:

$$SAR = \frac{C_V}{[Fe]} \Delta T \Big/ \Delta t \qquad (2)$$

Where C_V is the water specific heat, [Fe] is the Fe concentration in the colloid and $\Delta T/\Delta t$ is the initial slope (15s) of the heating curves of MNPs exposed to AMF.

Metal affinity conjugation of GFP and RFP to MNPs

sGFP and RFP conjugation was carried out by incubating 50 ug_{GFP} with 0.1 mg_{Fe}. in 20 mM HEPES buffer pH 8. The reaction was maintained under a continuous stirring at room temperature for 1 hour under protection from light. The particles were then separated with a magnet, dispersed in fresh buffer and stored at 4°C. The supernatant was collected for further analysis.

Protein concentration determination

The protein concentration was determined by Bradford assay using Bovine Serum Albumin (BSA, Sigma Aldrich, USA) as standard. Briefly,150µL of each standard or unknown sample were pipetted into the appropriate microplate wells. Then, 150µL of the Coomassie Plus Reagent (Thermofisher Scientific, USA) were added to each well and mixed for 30 seconds. The plate was incubated for 10 minutes at room temperature, then the absorbance at 595nm was measured using a microplate reader (ThermoFisher Scientific, USA).

Protein yield

The amount of protein bound to the carrier is expressed as the difference between the amount of protein offered in reaction and the amount of protein unbound in the solution (Equation 3).

$$P_{immob} = P_{offered} - P_{sup} \quad (3)$$

Where: P_{immob} represents the amount of protein bound to the MNPs, $P_{offered}$ is the amount of protein offered in reaction and P_{sup} is amount of unbound protein, found in the in the supernatant and the washing steps.

The immobilization yield (Y) was calculated as the ratio between the amount of protein immobilized on to the carrier and the amount of protein offered to the carrier (Equation 4).

$$Y(\%) = \frac{P_{immob}}{P_{offered}} \times 100 \quad (4)$$

Independent effect of global or local magnetic heating on conjugated sGFP or RFP fluorescence

The effect of global or local heating was assessed independently on the different selected MNPs functionalized with sGFP or RFP. The workflow followed was similar independently of the heating source used:

The samples were diluted to contain 10 µg/mL of sGFP or RFP and the effect of temperature on their fluorescence was studied. Each sample was incubated for 5 minutes: i) at different temperatures in a thermomixer under shaking at 350 rpm when global heating was applied, and ii) at different AMF settings in an AMF inductor (D5 AMF driver equipped with a CAL-2 coil-set, nB nanoScale Biomagnetics) when local magnetic heating was applied. In each case, after incubation, a 5-minutes cooling step in ice to stop heating was carried out. MNPs functionalized with sGFP or RFP incubated 5 min at 20ºC were used as controls non-exposed to heating.

To minimize the interference of iron oxide nanoparticles with the fluorescent signal of the nanothermometers, the fluorescent proteins were then eluted by incubation for 30 minutes with an excess of 0.5 M imidazole (Fluka, USA) and the MNPs were subtracted magnetically. In order to ensure MNPs extraction, the samples were additionally centrifuge for 5 minutes at 13000 rpm, the supernatant was collected and their protein content and fluorescence intensity

analyzed. The excitation and emission spectra of the samples were collected using a spectrofluorimeter (PerkinElmer, USA): i) excitation: 488 nm; emission: 510 nm in the case of sGFP; ii) excitation: 588 nm; emission: 610 nm in the case of RFP.

In all cases 90-100% elution of the bound GFP or RFP was observed. Fluorescence intensity of the eluted GFP or RFP was normalized with respect to the amount of eluted protein, even though the difference in protein content among the different eluted samples was minimal, and it is well known that the fluorescent proteins quenching in solution is insignificant even at high concentrations [10]. The percentage of relative fluorescence against the controls non-exposed to heating was then calculated. Local temperature reached during the application of the AMF was determined comparing the percentage of fluorescence intensity loss with the calibration curves obtained using the thermoblock as global heating source.

Combinatorial effect of the application of an alternating magnetic field to the conjugated GFP and RFP fluorescence

OP-RFP and TD-sGFP complexes were mixed in the same pot to obtain a suspension containing 10 µg mL-1 of each fluorescent protein. The local temperature registered from sGFP and RFP fluorescence in the mixture colloid after 5 min of exposure to AMF1=100 kHz - 50mT and AMF2=388 kHz - 10mT was estimated following the same workflow as when working with the complexes individually.

Complementary data

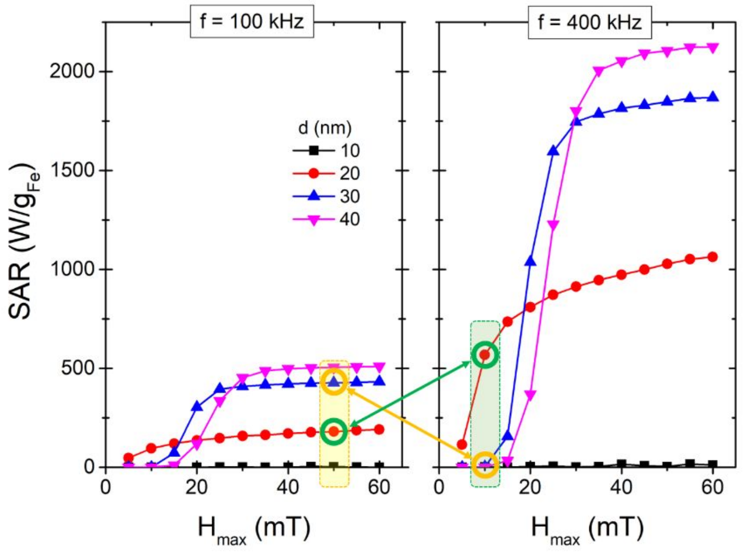

Figure S1. Theoretical specific absorption rate (SAR) of MNPs with different sizes exposed to low frequency (100 kHz) and high frequency (400 kHz) alternating magnetic fields (AMF) of increasing maximum field intensity (H_{MAX}).

The **Figure S2** display the X-ray diffraction patterns of the three samples used as nanoheaters in this study. All of them present the typical peaks of an inverse spinel and can be identified as magnetite. The width at half maximum results inverse to the size observed by TEM for each MNP.

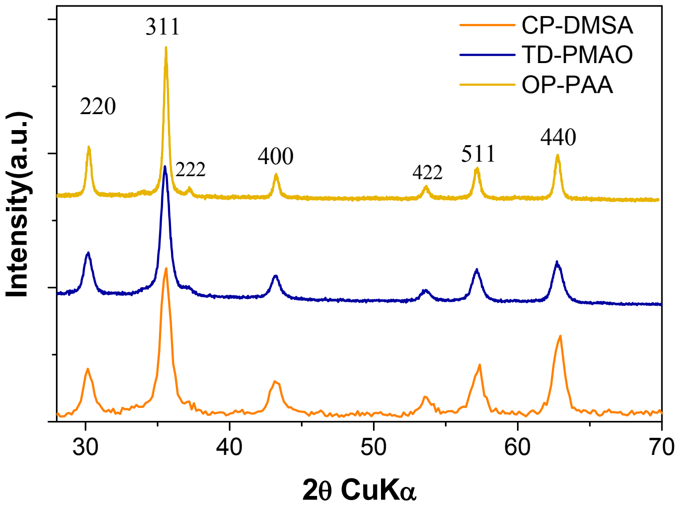

Figure S2. X-ray diffraction pattern of samples CP-DMSA, TD-PMAO and OP-PAA.

Table S1. Physicochemical properties of the MNPs synthesized: TEM average size (D_{TEM}), TEM distribution width (σ_{TEM}), hydrodynamic size (D_H), Polidispersity Index (PDI), ξ-potential (Z-pot), percentage of organic content (TG).

Sample	D_{TEM} (nm)	σ_{TEM}	D_H (nm)	PDI	Z-pot (mV)	TG (%)
CP-DMSA	*8.5*	*0.23*	*117*	*0.20*	*-36*	11.3
TD-PMAO	*20.2*	*0.23*	*89*	*0.25*	*-30*	26.1
OP-PAA	*33.2*	*0.23*	*97*	*0.17*	-44	3.4

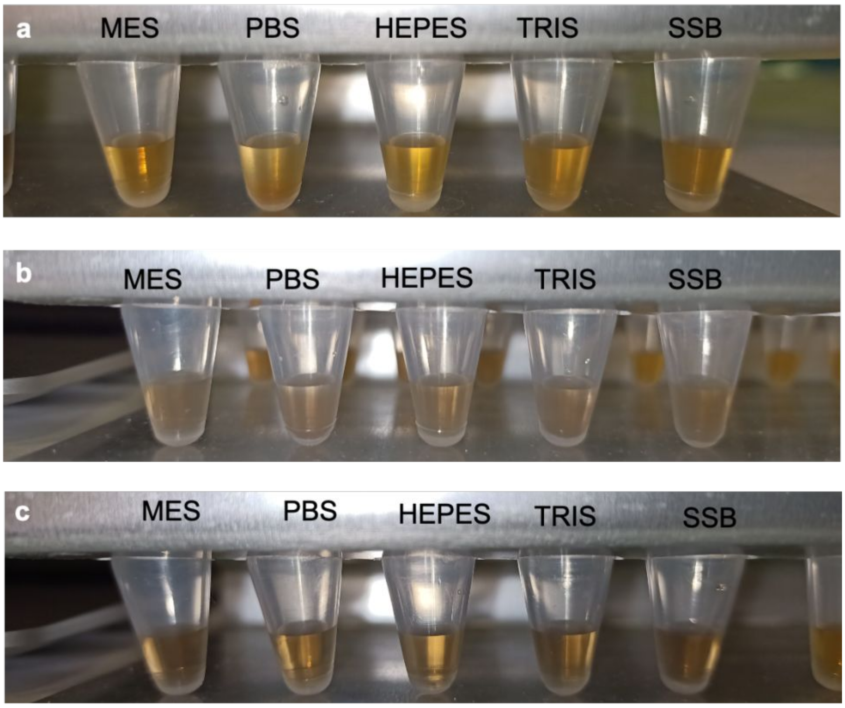

Figure S3. Colloidal stability of CP-DMSA (a), TD-DMSA (b) and OP-PAA (c) samples in different buffer solution: 10 mM MES pH7.0, PBS 1X, 20 mM HEPES pH 8.0, 20 mM TRIS pH 8 and 50 mM SSB pH 9.0.

In **Figure S4a**, the FT-IR spectra confirm the presence of carboxylic groups on the surface of the MNPs. The CP-DMSA spectrum shows two broad peaks at 1593 and 1376 cm^{-1} corresponding to asymmetric and symmetric stretch of COO^- groups of DMSA respectively. In the case of TD-PMAO, the sample presents a combination of a wide peak at 1400 cm^{-1} created by the bending modes of CH_2 of residual oleic acid trapped within PMAO chains with the 1551 and 1695 cm^{-1} sharp peaks generated by the carbonyl stretching modes. The OP-PAA coating

shows two pairs of resolved peaks at 1638/1568 cm^{-1} and 1544/1405 cm^{-1} that suggest the presence of both protonated and deprotonated carboxylic groups in the polymeric chains. The broad band at 600 cm^{-1} present in all the spectra is generated by the Fe-O groups of the magnetite/maghemite. TG curves in **Figure S4b** also show different trends for DMSA and polymeric coatings. The formers, present a continuous drop between 150 ºC and 400 ºC, whereas the laters show a sharper step around 280ºC. The resulting mass loss is significantly more pronounced in PMAO-coating (26.1 %) since it contains a double layer of organic mass made of oleic acid and PMAO crowns. In the case of CP-DMSA, the small size of the magnetic cores implies a large relative surface, and thus a mass loss of 11.3 % is reasonable compared to reported values. The smallest drop was observed for OP-PAA sample (3.4 %), indicating that the high-pressure homogenization conditions are able to generate a thin coating of PAA on the surface of MNPs, enough to stabilize the colloids.

Z-potential measurements are consistent with the presence of charged carboxylic groups on the surface of the MNPs. The three samples present negative Z-potentials below -25mV at neutral pH. The high surface charge observed for PAA coating (-40 mV), with just 3.4% of organic mass, points out the efficiency of the coating method employed (high pressure) to decorate the surface of large MNPs with multiple functional groups.

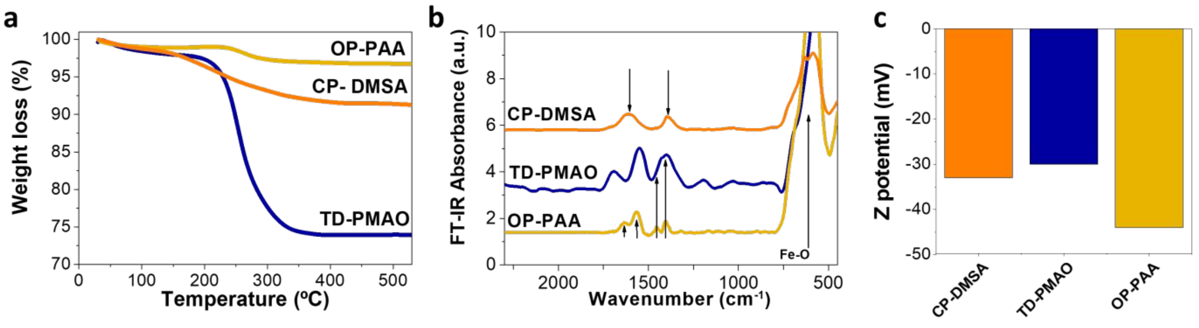

Figure S4. The surface of MNPs was decorated with carboxylic functional groups using organic and polymeric coatings of DMSA, PMAO and PAA. a) TG curves, b) FT-IR absorbance and c) Z-potential values for CP-DMSA, TD-DMSA and OP-PAA samples.

Table S2. Description of the proteins used as molecular thermometers at the MNP surface.

	GFP	mCherry
**Origin of protein sequence**	Aequorea victoria	Discosoma sp.
**Heterologous host**	E.coli	E.coli
**Tag**	His-tag	His-tag
**Mutations**	His-tag at N-terminus	His-tag at N-terminus
**Quaternary structure**	Monomeric	Monomeric
**Molecular weight**	29 kDa	30 kDa
**Isoelectric point**	6,37 (Theoretical)	6,01 (Theoretical)
**Extinction coefficient (280 nm) (M^{-1} x cm^{-1})**	19035 (Theoretical)	34380 (Theoretical)
**PDB ID**	1GFL	2H5Q

The effect of imidazole addition and of the incubation in ice on fluorescence was tested and no significant changes were observed in the GFP and RFP spectra at the conditions tested (**Figure S5**). Besides, the changes in fluorescence intensity after the heating treatment were stable upon time (at least 2 days for GFP and 5 days for RFP) after the protein elution from the MNPs. In addition, as it well known that GFP emission profile is more pH dependant compared to RFP [BMC Research Notes, 2010, 3, Nº: 303], the pH was monitored for each sample all along the incubation and elution process, and non-significant alterations were observed. All these controls support the fact that the changes observed on the fluorescent decay of the eluted proteins samples after the heating process could be used as a reporter of the temperature to which they were subjected.

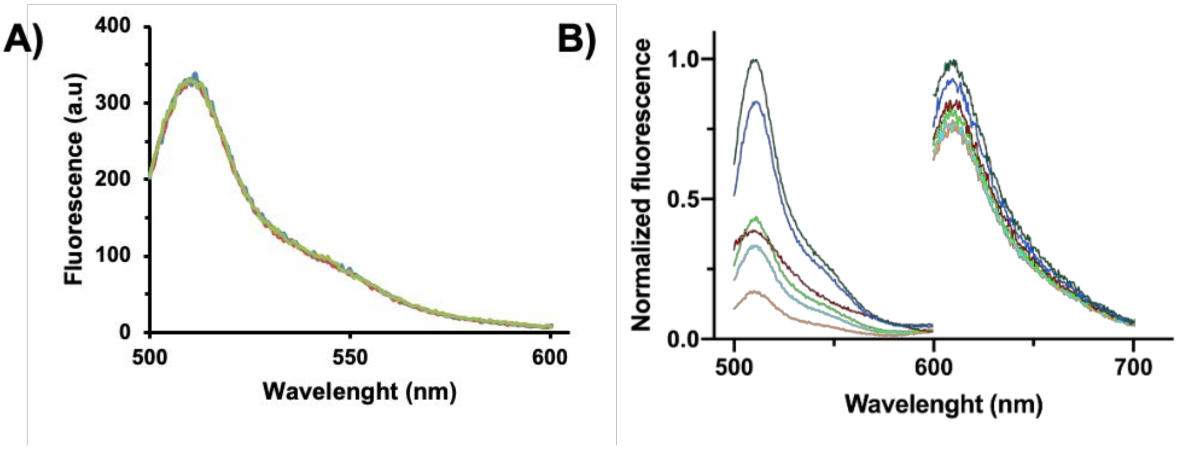

Figure S5. A) Fluorescence intensity of soluble GFP incubated at 5 minutes at 40ºC (blue line), incubated 5 minutes at 4ºC after the 40ºC incubation (red line) and incubated with 0.5 M imidazole for 30 minutes after the two previous incubations. B) Fluorescence intensity decay

of GFP and RFP after 5 days. Fluorescence of proteins incubated at time 0 are shown in dark green (20ºC), blue (50 ºC) and red (80ºC). The intensity of the same protein solution stored for 5 days at 4ºC is shown in light green (20ºC), light blue (50 ºC) and orange (80ºC).

Table S3. Fluorescence intensity vs. temperature calibration curves fittings for GFP and RFP, before and after immobilization on MNPs.

Sample	Linear fitting		Non-linear fitting					
	Slope	R^2	Fitting parameters					
			a	b	c	d	e	R^2
GFP	-0.5791	0.965	-	-	-	-	-	-
RFP	-0.7433	0.980	-	-	-	-	-	-
CP-GFP	-	-	9x10-6	-0.0026	0.2534	-10.536	228.92	0.999
CP-RFP	-1.35	0.987	-	-	-	-	-	-
TD-GFP	-1.2999	0.976	-	-	-	-	-	-
TD-RFP	-	-	-0.0005	0.101	-6.7127	198.07	-	0.9989
OP-GFP	-0.9279	0.980	-	-	-	-	-	-
OP-RFP	-0.9884	0.988	-	-	-	-	-	-

The suspension of 5 ug/mL of OP-GFP and 5 ug/mL of TD-RFP was prepared in water and incubated at 25 ºC to evaluate the colloidal stability of the individual colloids and the mix (OP-GFP + TD-RFP). The hydrodynamic diameter of the particles was monitored during time (**Figure S6**). Only one population of particles was observed in all the cases and no significant changes were observed during time.

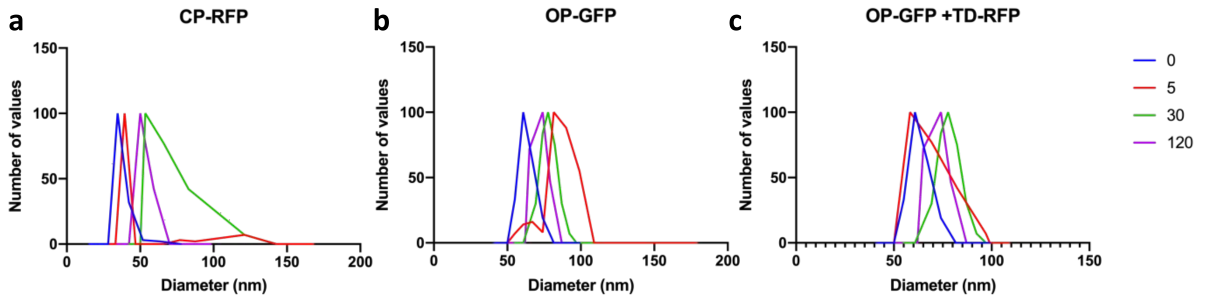

Figure S6. DLS meassurements of hydrodynamic size distribution over time (t=0, 5, 30 and 120 min) for: a) CP-RFP, b) OP-GFP and a) CP-RFP + OP-GFP.

SI References

[1] P. B. Balakrishnan, N. Silvestri, T. Fernandez-Cabada, F. Marinaro, S. Fernandes, S. Fiorito, M. Miscuglio, D. Serantes, S. Ruta, K. Livesey, O. Hovorka, R. Chantrell, T.

Pellegrino, *Adv. Mater.* **2020**, *32*, 2003712.
[2] M. J. Donahue, D. G. Porter, *OOMMF User's Guide, Version 1.0.*
[3] R. Massart, *Preparation of Aqueous Magnetic Liquids in Alkaline and Acidic Media*, Vol. 17, **1981**, pp. 1247–1248.
[4] R. Costo, V. Bello, C. Robic, M. Port, J. F. Marco, M. Puerto Morales, S. Veintemillas-Verdaguer, *Langmuir* **2012**, *28*, 178.
[5] N. Fauconnier, J. N. Pons, J. Roger, A. Bee, *J. Colloid Interface Sci.* **1997**, *194*, 427.
[6] M. Moros, B. Pelaz, P. López-Larrubia, M. L. García-Martin, V. Grazú, J. M. De La Fuente, *Nanoscale* **2010**, *2*, 1746.
[7] M. Marciello, V. Connord, S. Veintemillas-Verdaguer, M. A. Vergés, J. Carrey, M. Respaud, C. J. Serna, M. P. Morales, *J. Mater. Chem. B* **2013**, *1*, 5995.
[8] J. Teller, F. Westphal, C. Gruettner, *(Micromod Partikeltechnologie GmbH) - US7691285B2*, **2003**.
[9] I. Armenia, M. V. Grazú Bonavia, L. De Matteis, P. Ivanchenko, G. Martra, R. Gornati, J. M. de la Fuente, G. Bernardini, *J. Colloid Interface Sci.* **2019**, *537*, 615.
[10] M. C. Gather, S. H. Yun, *Nat. Commun.* **2014**, *5*, 5722.
